# Cost of illness of the prostate cancer in Japan—a time-trend analysis and future projections

**DOI:** 10.1186/s12913-015-1103-x

**Published:** 2015-10-05

**Authors:** Takefumi Kitazawa, Kunichika Matsumoto, Shigeru Fujita, Kanako Seto, Shimpei Hanaoka, Tomonori Hasegawa

**Affiliations:** Department of Social Medicine, Toho University School of Medicine, 5-21-16, Omori-nishi, Ota-ku, 143-8540 Tokyo Japan; Chiba Psychiatric Medical Center, Chiba, Japan

**Keywords:** Cost of illness, Prostate cancer, Health economics, Health policy

## Abstract

**Background:**

The majority of patients with prostate cancer (International Classification of Diseases, 10th edition, code C61) are elderly. With Japan’s rapidly society aging, both the prevalence and mortality of prostate cancer are expected to increase in the future. The objective of this study was to estimate and predict the cost of illness (COI) associated with prostate cancer in Japan.

**Methods:**

Using a COI method based on available data from government office statistics, we estimated the COI for 2002, 2005, 2008, and 2011. We then predicted the COI for 2014, 2017, and 2020 using fixed model estimation and variable model estimation. With fixed model estimation, only estimated future population was used as a variable. Variable model estimation considered the time trend of health-related indicators in the past 15 years. We derived the COI from the sum of direct and indirect costs (morbidity and mortality).

**Results:**

We found the predicted future COI of prostate cancer to be 354.7–378.3 billion yen in 2014, 370.8–421.0 billion yen in 2017, and 385.3–474.1 billion yen in 2020. Regardless of the estimation model, we found that COI would increase compared with the baseline year 2011 (307.3 billion yen). The direct costs for inpatient and outpatient treatment, laboratory tests, and drugs accounted for 60–75 % of the COI of prostate cancer.

**Conclusions:**

The results of this study suggest that the COI of prostate cancer in Japan has steadily increased and is expected to rise in the future. Direct costs comprised the largest proportion of the COI and are anticipated to continue expanding; this will result in increased burden on public funds in Japan, where a universal public insurance system operates. These trends differ from those with other forms of cancer.

## Background

Prostate cancer is one of the most common cancers in men, particularly in the United States, Canada, and Europe. The prevalence and incidence of prostate cancer varies across countries [1, 2]. In recent years, patient numbers and mortality rates for prostate cancer (International Classification of Diseases, 10th edition, code C61) have increased in Japan. Prostate cancer is the seventh-leading cause of cancer death in Japanese men. The majority of prostate cancer cases involve the elderly. According to the governmental Patient Survey [3], 60.8 % of prostate cancer patients were 75 years or older; this compares with 42.3 % for lung cancer and 37.5 % for stomach cancer. The average age of death from prostate cancer in Japan in 2008 was 78.8 years, which was higher than with other cancers, such as lung (74.1 years) and stomach cancer (74.4 years). As Japan’s elderly population continues to grow, the prevalence and mortality of prostate cancer are expected to increase proportionately.

Age-standardized incidence of prostate cancer is high in the United States and Canada [4]. Studies that have attempted to clarify the social burden of prostate cancer have also been conducted in those countries [5]. Roehrborn et al. [6] researched the cost of prostate cancer in several countries, including the United States. They suggested that despite declining mortality rates, the social burden of prostate cancer in the United States would continue to increase owing to a greater number of patients being diagnosed and treated at an earlier stage. Medical expenses for the treatment of prostate cancer in Canada were estimated from a social perspective [7]. It was found that the direct health-care costs of treating over 700,000 cases of prostate cancer among Canadian men aged 40–80 years in 1997 would amount to C$9.76 billion. The social burden of prostate cancer—including productivity loss from premature death and the many costs associated with morbidity—have been fully investigated [8]. To determine the social burden of prostate cancer in Japan, assessments based on the Survey of Medical Care Activities in Public Health Insurance and the Patient Survey have been conducted [9]; however, most of those were snapshot estimations that did not consider demographic dynamics, such as Japan’s rapidly aging population.

A comprehensive economic analysis demands a consideration of both direct and indirect costs; the latter include productivity losses as a result of individuals unable to work because of hospitalization or outpatient visits, as well as premature death resulting from the illness. The cost of illness (COI) method developed by Rice and co-workers [10, 11], which evaluates financial losses as a result of illness in a prescribed statistical universe, has been widely used to estimate the social burden of disease. The COI can be used as a criterion for decision making in allocating limited budgets and resources for governmental health policies in disease control. By applying this approach with diseases, it is possible to compare and set priorities in policy making for the simultaneous management of several major diseases.

The purpose of this study was to estimate and predict the economic burden associated with prostate cancer in Japan compared with other cancers.

## Methods

We used the COI method to examine the economic burden of prostate cancer. Calculation method employed in this study which used government office statistics and the COI method was same as our previous study [12]. First, we calculated COI of 2002, 2005, 2008, and 2011. We then predicted the future COI [13] based on those results. We adapted top-down approach of COI method aggregating published national surveys and based on prevalence of prostate cancer.

### Data sources

We used the government office statistics listed below. The survey results of the Central Social Insurance Medical Council were employed to determine the hospitalization cost per day of prostate cancer. The Survey of Medical Care Activities in Public Health Insurance was used to examine outpatient cost per day. We used the Basic Survey on Wage Structure, Labour Force Survey, and Estimates of Monetary Valuation of Unpaid Work to calculate labour value. We employed Vital Statistics to evaluate the number of deaths caused by prostate cancer. The Patient Survey was used to identify the number of patients, total person-days of outpatient visits, and average length of stay for prostate cancer. Population Projections for Japan released by the National Institute of Population and Social Security Research in Japan were used to refer future population.

### Measurement and valuation

Components of COI were shown in Table 1. The COI comprises direct cost (DC) and indirect cost (IC). IC includes morbidity cost (MbC) and mortality cost (MtC). COI is calculated using the following equation:

$$ \mathrm{C}\mathrm{O}\mathrm{I}=\mathrm{D}\mathrm{C}+\mathrm{M}\mathrm{b}\mathrm{C}+\mathrm{M}\mathrm{t}\mathrm{C}. $$

**Table 1:** Components of COI

Components of COI				Formulas / Data sources
COI			Cost of illness	DC + MbC + MtC
DC			Direct cost	HC + OC
	HC		Hospitalization cost	iCd × THD
		iCd	Inpatient cost per day	Survey results of the Central Social Insurance Medical Council
		THD	Total person-days of hospitalization	Patient Survey
	OC		Outpatient cost	oCd × TOVy
		oCd	Outpatient cost per day	Survey of Medical Care Activities in Public Health Insurance
		TOVy	Total person-days of outpatient visits	Patient Survey
MbC			Morbidity cost	MbCi + MbCo
	MbCi		Morbidity cost of inpatients	THD × LVd
		THD	Total person-days of hospitalization	Patient Survey
		LVd	1-day labour value per person	Basic Survey on Wage Structure, Labour Force Survey, Estimates of Monetary Valuation of Unpaid Work
	MbCo		Morbidity cost of outpatients	TOVy × LVd/2
		TOVy	Total person-days of outpatient visits	Patient Survey
		LVd/2	1/2-day labour value per person	Basic Survey on Wage Structure, Labour Force Survey, Estimates of Monetary Valuation of Unpaid Work
MtC			Mortality cost	NDy × LVl
	NDy		Number of deaths	Vital Statistics
	LVl		Lifetime labour value per person	Basic Survey on Wage Structure, Labour Force Survey, Estimates of Monetary Valuation of Unpaid Work, Life table

The DC comprises hospitalization cost (HC) and outpatient cost (OC) and it is calculated using the following equation:

$$ \mathrm{D}\mathrm{C}=\mathrm{H}\mathrm{C}+\mathrm{O}\mathrm{C}=\mathrm{i}\mathrm{C}\mathrm{d}\times \mathrm{T}\mathrm{H}\mathrm{D}+\mathrm{o}\mathrm{C}\mathrm{d}\times \mathrm{T}\mathrm{OVy}. $$

The HC was determined by multiplying the inpatient cost per day (iCd) for prostate cancer and total person-days of hospitalization (THD). The OC was determined by multiplying the OC per day (oCd) for all cancers and the total person-days of outpatient visits (TOVy) for prostate cancer.

All inpatients were assumed to undergo an operation, but the cost of radiotherapy was not included. Because data were available only for 2005, iCd of 2005 (48,844 yen) was used as the baseline. The iCd of prostate cancer was calculated assuming the rate of change of iCd for prostate cancer to be the same as that of iCd for all cancers. The iCd for each year was calculated by multiplying the rate of change and the value for 2005. Watchful waiting is one of the major treatments in Japan. Patients who underwent watchful waiting were included in TOVy. The oCd was 19,731 yen in 2011.

MbC comprised the MbC of inpatients (MbCi) and outpatients (MbCo). MbCi was calculated by multiplying 1-day labour value per person (LVd) and the THD according to sex and 5-year age groups. MbCo was determined by multiplying the half of LVd and TOVy according to sex and 5-year age groups.

MtC was measured as the loss of human capital (human capital method), which was calculated by multiplying the number of deaths (NDy) and the lifetime labour value per person (LVl). LVl was determined by summing the patients’ potential income, which would have accrued in the future had they not died, from the age of death to life expectancy. MbC and MtC were calculated using the following equations:

$$ \mathrm{M}\mathrm{b}\mathrm{C}=\mathrm{MbCi}+\mathrm{MbCo}=\mathrm{T}\mathrm{H}\mathrm{D}\times \mathrm{L}\mathrm{V}\mathrm{d}+\mathrm{TOVy}\times \mathrm{L}\mathrm{V}\mathrm{d}/2. $$

$$ \mathrm{M}\mathrm{t}\mathrm{C}=\mathrm{N}\mathrm{D}\mathrm{y}\times \mathrm{L}\mathrm{V}\mathrm{l}. $$

Pensions were not included in MbC and MtC. Future labour value was adjusted to a present value using a 3 % discount rate.

### Statistical approach

To examine changes over time, we first estimated the COIs for 2002, 2005, 2008, and 2011 using available data. Next, to make future predictions, we estimated the COIs for 2014, 2017, and 2020 using the two methods described below. The Patient Survey is conducted every 3 years in Japan. We calculated the COI to match the year of the Patient Survey.

Estimates of future COI were made using two methods [13]—fixed model estimation and variable model estimation. Variable model estimation consisted of a linear model, logarithmic model, and mixed model depending on which formula was selected for the health-related indicators.

Fixed model is the estimation that assumed health-related indicator (such as mortality rate, number of outpatient visits, and average length of stay) were fixed and it unrelated to the time trend of health-related indicators. We used those values at 2011, and only future population estimation was used as a variable. With fixed model estimation, we first calculated the mortality rate, number of outpatient visits per population, and number of hospitalizations per population according to sex and 5-year age groups in 2011 as standard year indicators. Next, by multiplying these factors with future population estimates according to sex and 5-year age groups for 2014, 2017, and 2020, we calculated the predictive number of deaths, TOVy, and THD. We estimated MbC and MtC for 2014, 2017, and 2020 using the 2011 data for average length of stay, life expectancy, labour value, iCd, and outpatient visits.

With variable model estimation, we drew a trend line for each health-related indicator by using a logarithmic or linear approximation with six time points referencing 1996, 1999, 2002, 2005, 2008, and 2011. We then extended the trend line for each approximation to determine values for 2014, 2017, and 2020. Variable models assumed every health-related indicator as a variable. Indicators had their own trend line. Difference of time trend among indicators was reflected in calculation process by variables. This is the reason why it was named variable model estimation for this approach.

The estimated future health-related indicators were used in addition to future population estimates. Because the trend of each health-related indicator was different, the single model estimation (logarithm model and linear model) might not predict future COI precisely. Therefore, we developed a mixed model, which adopted the value with a higher coefficient of determination for each 5-year age groups [13]. Our mixed model was an approximation using health-related indicators with a higher coefficient of determination in a logarithmic and linear approximation for each 5-year age groups. By mixing the optimal results of both logarithmic and linear model depending on the characteristics of each health-related indicator, getting more appropriate estimates could be possible. This is the reason why it was named mixed model for this approach. This method was already employed in our previous study [13]. Because the mixed model was a combination of models with a higher coefficient of determination, we believed the mixed model to be the most valid for this study. The fixed model was the simplest method and could be regarded as a reference. The estimations using the logarithmic and linear models can be regarded as a sensitivity analysis to test the robustness of the mixed model.

When estimating using the trend line, a future predicted value sometimes took less than 0. They are unlikely to reflect actual conditions. Therefore, we needed to set a “minimum value”. For mortality rate, number of times of outpatient visit per population and number of times of hospitalization per population, the minimum value was set as the value from the year prior to that in which the estimate was less than 0. In this study, we assumed that the value from the year prior to that when the estimate was less than 0 would be maintained thereafter. Negative value was not used in this model. As for average length of stay, we set 8.1 days the average length of stay of patients with prostate cancer (2012) of 28 Organization for Economic Co-operation and Development (OECD) countries - as “minimum value”.

The current study used only anonymous governmental data; no human or animal subjects were involved. No institutional review is required for this type of research in Japan [14].

## Results

### Estimates of health-related indicators

In conceiving this study, we expected that the number of deaths would increase across all models. With a reference value of 1.00 for 2011, we found that the values would increase as follows: to 1.15 (fixed model) and 1.20 (logarithmic model) in 2014; 1.27 (fixed model) and 1.33 (logarithmic model) in 2017; and 1.37 (fixed model) and 1.44 (logarithmic model) in 2020.

We found that the number of outpatients would also increase proportionately. With a reference value of 1.00 in 2011, the values would increase as follows: to 1.09 (fixed model) and 1.36 (linear model) in 2014; 1.10 (fixed model) and 1.55 (linear model) in 2017; and 1.20 (fixed model) and 1.80 (linear model) in 2020. The total number of times of hospitalization and THD would also increase (Table 2).

**Table 2:** Projected results of health-related indicators of prostate cancer*

	2011 (base line)			Future estimates					
				2014		2017		2020	
Number of prostate cancer death (person)			Fixed model	12,425	(1.15)	13,726	(1.27)	14,833	(1.37)
	10,823	(1.00)	Linear model	12,862	(1.19)	14,165	(1.31)	15,352	(1.42)
			Logarithmic model	12,934	(1.20)	14,341	(1.33)	15,597	(1.44)
Number of outpatient visit (person)			Fixed model	5,585,678	(1.09)	5,655,557	(1.10)	6,176,422	(1.20)
	5,139,223	(1.00)	Linear model	6,979,937	(1.36)	7,987,933	(1.55)	9,254,791	(1.80)
			Logarithmic model	5,696,500	(1.11)	6,154,510	(1.20)	6,747,682	(1.31)
Number of times of hospitalization (times)			Fixed model	15,061	(1.05)	15,489	(1.08)	15,911	(1.11)
	14,300	(1.00)	Linear model	17,981	(1.26)	20,439	(1.43)	23,251	(1.63)
			Logarithmic model	15,673	(1.10)	16,822	(1.18)	18,155	(1.27)
Total days of hospitalization (days)			Fixed model	1,938,841	(1.10)	2,069,475	(1.18)	2,178,139	(1.24)
	1,756,560	(1.00)	Linear model	1,769,365	(1.01)	2,011,227	(1.14)	2,287,945	(1.30)
			Logarithmic model	1,895,770	(1.08)	1,884,734	(1.07)	1,869,085	(1.06)

*Index number having a value of 1.00 at baseline shown in parentheses

We found that the average age of death rose from 77.9 years (2002) to 79.3 years (2011). This trend would continue in the future, rising as follows: to 79.9 years (fixed model) and 80.3 years (logarithmic model) in 2014; 80.4 years (fixed model) and 80.8 years (linear model) in 2017; and 80.7 years (fixed model) to 81.3 years (linear model) in 2020. The estimated average age of death in 2014 with the logarithmic model was higher than with the linear model. However, the estimated average age of death in 2017 and 2020 with the linear model was higher than with the logarithmic model. The linear model displayed a higher slope between 2014 and 2020 than the logarithmic model (Table 3). The value of the average length of stay in 2020 estimated using the linear model was less than 0.

**Table 3:** Average age of death from prostate cancer

	2002	2005	2008	2011		Future estimates		
						2014	2017	2020
Average age of death (years)					Fixed model	79.9	80.4	80.7
	77.9	78.3	78.8	79.3	Linear model	80.2	80.8	81.3
					Logarithmic model	80.3	80.7	81.1

### Estimates of COI

The estimated COI of prostate cancer was 174.5 billion yen in 2002, 246.9 billion yen in 2005, 286.0 billion yen in 2008, and 307.3 billion yen in 2011. The future estimates for the COI appear in Table 4. The health-related indicators used for the future estimates are presented in Table 2. The COI would increase as follows: to 354.7 billion yen (fixed model) and 378.3 billion yen (linear model) in 2014; 370.8 billion yen (logarithmic model) and 421.0 billion yen (linear model) in 2017; and 385.3 billion yen (logarithmic model) and 474.1 billion yen (linear model) in 2020. With the mixed model, a logarithmic approximation was used for the average length of stay and a linear approximation was also employed for the number of outpatient visits, hospitalizations per population, and mortality. With the mixed model, the COI was estimated to increase from 362.0 billion yen in 2011 to 451.9 billion yen in 2020—a 1.5-fold increase.

**Table 4:** Results of estimated COI of prostate cancer (billion yen)

		2002	2005	2008	2011		Future estimates		
							2014	2017	2020
Direct cost		97.0	164.2	195.9	219.4	Fixed model	240.4	250.6	268.1
						Linear model	256.5	292.7	336.2
						Logarithmic model	239.7	248.0	258.6
						Mixed model	256.5	292.7	336.2
	Inpatient care	66.3	96.1	115.4	118.0	Fixed model	130.2	139.0	146.3
						Linear model	118.8	135.1	153.6
						Logarithmic model	127.3	127.0	125.5
						Mixed model	118.8	135.1	153.6
	Outpatient care	30.7	68.1	80.5	101.4	Fixed model	110.2	111.6	121.9
						Linear model	137.7	157.6	182.6
						Logarithmic model	112.4	121.4	133.1
						Mixed model	137.7	157.6	182.6
Mortality cost		66.9	65.8	72.7	70.0	Fixed model	95.6	100.9	104.9
						Linear model	96.6	100.2	105.9
						Logarithmic model	94.9	100.9	103.6
						Mixed model	79.5	81.6	83.6
Morbidity cost		10.7	16.9	17.5	17.9	Fixed model	18.7	19.4	19.4
						Linear model	25.2	28.2	31.9
						Logarithmic model	21.2	21.9	23.0
						Mixed model	26.0	28.7	32.1
COI		174.5	246.9	286.0	307.3	Fixed model	354.7	370.9	392.4
						Linear model	378.3	421.0	474.1
						Logarithmic model	355.8	370.8	385.3
						Mixed model	362.0	403.0	451.9

DC was the largest component of the COI, accounting for 55.6 % in 2002 and 71.4 % in 2011. With the mixed model, DC was expected to increase to 74.4 % in 2020. However, the mixed model predicted that the proportion of MtC would decrease from 22.8 % in 2011 to 18.5 % in 2020.

## Discussion

The COI of prostate cancer in Japan is expected to continue increasing. This study found that this trend will continue until at least 2020 and that DC will also steadily rise well into the future.

Because most prostate cancer patients are elderly, the COI of prostate cancer may differ from that with other cancers. Our previous study suggested that, in Japan, MtC accounted for 60–80 % of the COI in other forms of cancer, such as that of the stomach, colon, rectum, liver, lung, breast, uterus, malignant lymphoma, and leukaemia [12]. With prostate cancer, the MtC ranged from 25 to 38 %. The DC of the above-mentioned cancers accounted for 12–19 % of the COI; it was 56–69 % in prostate cancer. Such differences were owing to the average patient age and average age of death from prostate cancer being higher than those with other cancers. These differences have an effect on the time trend with the COI. Our previous study of the COI of stomach cancer in Japan [13] established that this COI exhibited a downward trend from 1996 to 2008 and that this would continue until 2020; this is thus the reverse of the trend with prostate cancer.

The human capital method evaluates the labour value of the elderly as low compared with that of younger people. Therefore, the opportunity cost of the elderly, which reflects hospitalization and outpatient visits as well as estimated income that patients might have earned during their working years, is projected to be lower than in younger people. The average age of death from cancers other than prostate was found to be 64.9–75.1 years [12] compared with 78.8 years for prostate cancer in 2008. According to our mixed model estimates, MtC increases were statistically lower than the rise in mortality. Therefore, the influence of increased MtC associated with the growing number of deaths from prostate cancer would be negligible in future valuations of the COI.

The tendency of a higher average age of prostate cancer patients and a higher average age of death will continue in the future; the proportion of mortality and morbidity costs in the COI of prostate cancer will remain low compared with that for other cancers. In countries such as Japan where public funds are used in medical insurance systems, an increase in the DC over time signifies that the public sector will continue to be burdened by expanding health-care costs. When evaluating the allocation of resources for cancer control in the future, it is necessary to consider differences in the characteristics among the different forms of cancer.

Several related studies on the social burden of prostate cancer have been published. Koinuma [9] reported that the cost of prostate cancer in Japan amounted to 270.9 billion yen in 2005; this result is similar to that in the present study (246.9 billion yen in 2005). His estimates included lost profits associated with morbidity and mortality as well as the health-care costs of prostate cancer using the Vital Statistics and the Patient Survey. That study’s findings were rudimentary since no time-trend analysis and future projection was performed.

Based on predictions from the National Cancer Center, it is estimated that deaths from prostate cancer in Japan will amount to 14,700 per year for 2020–2024 [15]. In this study, we estimated that the number of deaths from prostate cancer in 2020 would be 14,833 (fixed model) and 15,597 (logarithmic model).

The present study is not free of limitations. First, we used data obtained over a relatively short period (1996–2008) to predict health-related indicators; thus, caution should be applied when interpreting the results. For example, we estimated that the average age of death would continue to rise, reaching around 81 years in 2020. Such a prediction may be too high. The appropriateness of setting a minimum value should also be examined. The average length of stay for prostate cancer patients shortened drastically from 38.0 days in 1996 to 10.1 days in 2011; this occurred following health-care reforms, which included a change in the reimbursement scheme for hospitals. In this study, we set the minimum value for an average length of hospital stay at 8.1 days, which reflected the average value for OECD countries. Because the average length of stay may become shorter in the future, it is unclear whether or not this minimum value is appropriate. When interpreting future COI estimations for prostate cancer, these methodological limitations should be considered.

Second, this study has limitations related to data availability. Because we lacked data about alternative treatments, we assumed that all the hospitalized patients underwent surgery. Medical expenses could vary with endocrine and radiation therapies. The Ministry of Health, Labour and Welfare allows researchers to use nationwide claims data on public medical insurance; however, there are limits on the accessible years of inpatient data, and no representative cost data were available for our study.

Our estimation has no standard errors or confidence intervals. However, our study attempted multiple approaches. The fixed model could be regarded as a reference because it was the simplest estimation. The logarithmic model is the low-end (in 2017, 2020), and the linear model is the high-end estimation. This range of estimation would complement standard errors or confidence intervals.

One of the purposes of predicting the future COI is to provide basic information in allocating limited resources. In Canada, the government has calculated the COI of major diseases and used the findings to update its health-care policy [16]. Predicting the future COI of major diseases could similarly help in prioritizing and determining health-care policy in Japan.

## Conclusions

The results of this study suggest that the COI of prostate cancer in Japan has been increasing and will maintain this rise in the future. DC accounts for the largest proportion of COI and is expected to continue increasing; this will result in a further burden on public funds in Japan, which has a universal public insurance system. These trends are different from those observed with other cancers. When evaluating the allocation of resources for cancer control, it is necessary to consider differences in the characteristics of the forms of cancer.

## Abbreviations

COI: Cost of illness
DC: Direct cost
HC: Hospitalization cost
IC: Indirect cost
iCd: Inpatient cost per day
LVd: 1-day labour value per person
LVl: Lifetime labour value per person
MbC: Morbidity cost
MbCi: Morbidity cost of inpatients
MbCo: Morbidity cost of outpatients
MtC: Mortality cost
NDy: Number of deaths
OC: Outpatient cost
oCd: Outpatient cost per day
THD: Total person-days of hospitalization
TOVy: Total person-days of outpatient visits
